# Functional Potential of Peruvian Fava Bean Flours in Bread: Antioxidant Activity and Phenolic Bioaccessibility

**DOI:** 10.1007/s11130-026-01487-z

**Published:** 2026-03-21

**Authors:** Alessandra Andréa Pereira Nicolau, Rebeca Salvador-Reyes, Maria Teresa Pedrosa Silva Clerici, Bruno Martins Dala-Paula

**Affiliations:** 1 Faculdade de Nutrição, Universidade Federal de Alfenas (UNIFAL-MG), Alfenas, MG Brazil; 2 Departamento de Ciência de Alimentos e Nutrição, Faculdade de Engenharia de Alimentos, Universidade Estadual de Campinas, Campinas, SP Brazil; 3 Facultad de Ingeniería, Universidad Tecnológica del Perú, Lima, Peru; 4 Departamento de Alimentos, Faculdade de Farmácia, Universidade Federal de Minas Gerais (UFMG), Belo Horizonte, MG Brazil

**Keywords:** Antioxidants, *Vicia fava* L., Pulse, Baking, Sustainability

## Abstract

Plant-based foods are increasingly valued for their health-promoting and sustainable attributes. Among legumes, fava bean (*Vicia faba *L*.*) is notable for its nutritional density and bioactive compounds. This study evaluated the effect of partially substituting wheat flour with raw flours from three Peruvian cultivars (Verde, Quelcao, and Peruanita) on antioxidant potential and phenolic bioaccessibility in bread. Six bread formulations were prepared by replacing 10 and 20% of wheat flour with fava bean flours, alongside a control (100% wheat flour). Antioxidant activity was assessed using ABTS•⁺ and DPPH assays, while total phenolic content (TPC) and total flavonoid content (TFC) were measured spectrophotometrically. Phenolic bioaccessibility and antioxidant activity were further examined through *in vitro* gastrointestinal digestion following the INFOGEST protocol. Compared with the control, fava bean flours showed significantly higher antioxidant potential (ABTS 26.13 µmol TE/g; DPPH 0.90 µmol TE/g), TPC (1.61 mg GAE/g), and TFC (0.80 mg CE/g), with Peruanita achieving the highest values. Partial substitution enhanced the antioxidant properties of breads, particularly at 20%. After digestion, both breads and flours exhibited greater antioxidant potential and phenolic bioaccessibility, suggesting the release of bound compounds. Partial substitution of wheat flour with Peruvian fava bean flours improves bread’s nutritional and functional properties by enhancing antioxidant potential and phenolic bioaccessibility. These results underscore the value of underutilized Andean legumes as sustainable ingredients for developing functional bakery products with added health benefits.

**Supplementary Information:**

The online version contains supplementary material available at 10.1007/s11130-026-01487-z.

## Introduction

Plant-based diets, rich in whole grains, legumes, vegetables, and fruits, have been shown to provide health benefits while promoting environmental sustainability, contributing to increased human longevity [1]. Legumes are the primary plant-based sources of protein, and fava bean (*Vicia faba* L.) stands out for its nutritional profile and functional properties, including cholesterol reduction, glycemic control, and antioxidant activity [2]. Moreover, fava bean cultivation offers environmental advantages, such as soil restoration and the promotion of sustainable agriculture [3].

The growing interest in healthy foods has driven innovations in the baking sector, including the trend of replacing refined flours with legume-based whole flours, as suggested by Boukid et al. [4], can enhance the nutritional profile of processed foods by increasing their antioxidant potential, given their richness in phenolic compounds [5]. Bread is a staple food widely consumed worldwide, characterized by its affordability and economic production. The diversification of bread production reflects both consumer preferences and the demands associated with population growth [6].

Furthermore, the bioaccessibility of bioactive compounds, such as polyphenols and antioxidants, is essential to ensure their health benefits [7]. In vitro studies and digestion simulation methods, such as the INFOGEST protocol, are crucial to understanding how these compounds are released and absorbed in the human body, ultimately influencing the nutritional effectiveness of bakery products [8, 9].

**Fig. 1:**
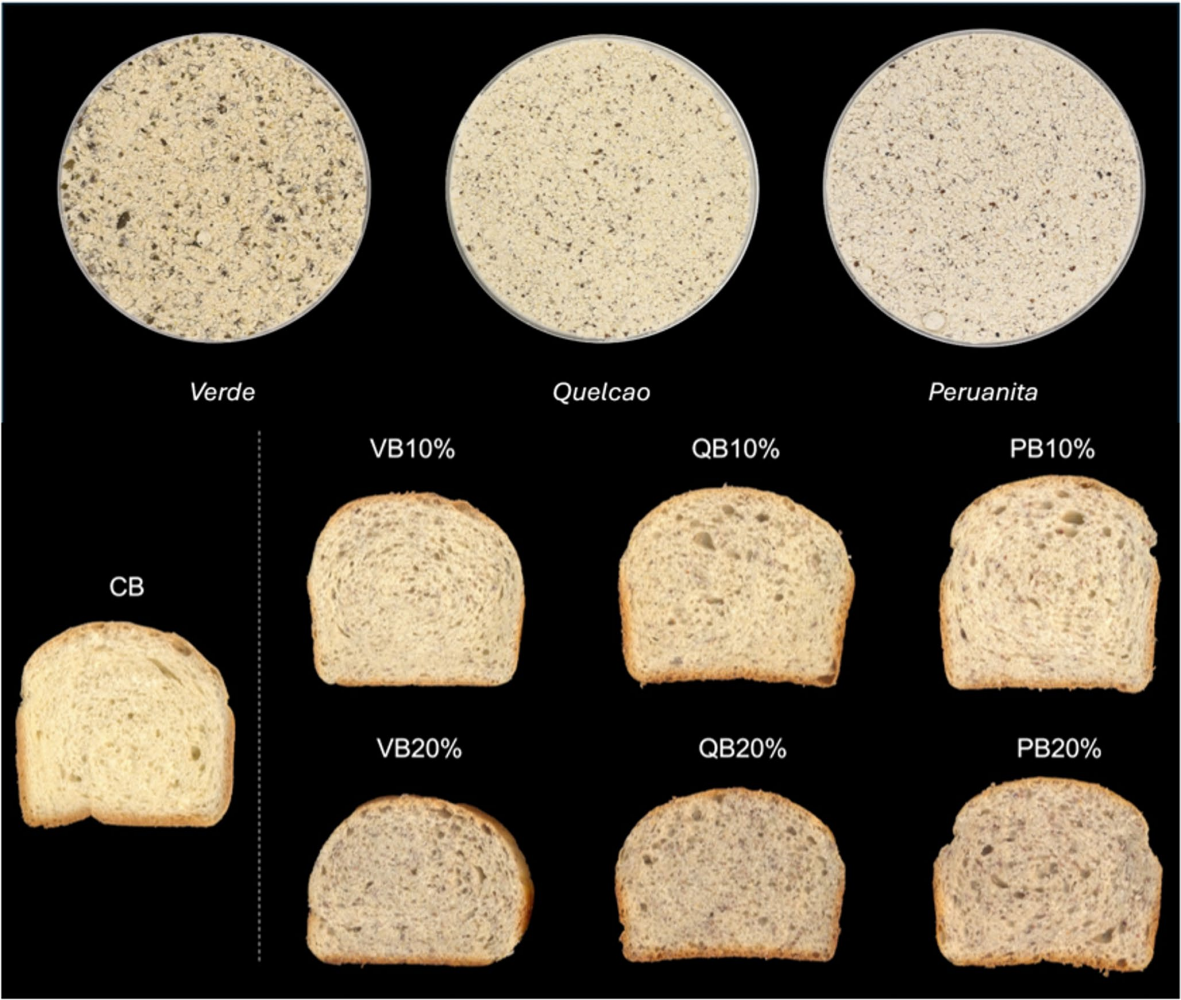
Flours from three Peruvian fava bean cultivars (*Verde*, *Quelcao*, and *Peruanita*) and bread formulations with partial substitution of wheat flour (10 and 20%) with these fava bean flours, compared with the control bread (100% wheat flour)

Recent work from our group has contextualized the Peruvian fava bean within Andean agrobiodiversity, underscoring its nutrient density and reported applications in cereal-based products, including bread [10]. Complementarily, the cultivar-level characterization of three representative Peruvian fava beans (Verde, Quelcao, and Peruanita) reported phenolic profiles and techno-functional properties relevant to breadmaking [2]. Together, this evidence supports the partial replacement of wheat flour with raw Peruvian fava bean flours to modulate dough properties while contributing phenolics and antioxidant potential to the final product. Accordingly, this study evaluates the preservation and bioaccessibility of phenolic compounds and the antioxidant potential in breads formulated by partially substituting wheat flour (10 or 20%) with raw flours from *Verde*, *Quelcao*, and *Peruanita* (Fig. 1).

## Materials and Methods

The materials and detailed procedures are described in the Supplementary Material.

## Results and Discussion

### Antioxidant Potential, Total Phenolic Content (TPC), and Total Flavonoid Content (TFC) in Raw Flours of Peruvian Bean Cultivars

Wheat flour exhibited the lowest antioxidant potential values in both methods employed (ABTS•+ and DPPH radical scavenging), when compared with the Peruvian bean flours (Table 1). The DPPH and ABTS•+ assays evaluate antioxidant capacity based on free radical scavenging activity, with the DPPH method generally being more stable and less sensitive to hydrophilic compounds than ABTS•+ [11]. In this study, Peruvian bean flours demonstrated higher antioxidant potential (by both methods) than wheat flour, with the *Peruanita* cultivar presenting the highest values. In the ABTS method, *Peruanita* flour exhibited approximately 19 times greater antioxidant potential than wheat flour. The *Verde* and *Quelcao* cultivars also showed higher values compared to wheat flour, with differences of approximately 17-fold.

**Table 1:** Antioxidant activity (ABTS and DPPH assays), total phenolic content, and flavonoid content of raw flours and breads prepared with Peruvian fava bean (*Vicia faba*) from diferent cultivars

Sample	Antioxidant potential (µmol TE/g)		TPC (mg EAG/g)	TFC (mg EC/g)
	ABTS	DPPH		
Flours				
WF	1.33 ± 0.09^c^	0.22 ± 0.03^d^	0.30 ± 0.02^c^	0.40 ± 0.02^b^
VF	23.78 ± 0.99^b^	0.55 ± 0.04^c^	1.40 ± 0.09^b^	0.79 ± 0.02^a^
QF	24.05 ± 0.73^b^	0.77 ± 0.54^b^	1.50 ± 0.09^b^	0.78 ± 0.03^a^
PF	26.13 ± 0.93^a^	0.90 ± 0.10^a^	1.61 ± 0.09^a^	0.80 ± 0.03^a^
Breads				
CB	1.25 ± 0.10^e^	0.21 ± 0.05^c^	0.35 ± 0.07^d^	0.35 ± 0.04^b^
VB 10%	1.87 ± 0.12^d^	0.20 ± 0.10^c^	0.49 ± 0.04^c^	0.37 ± 0.03^b^
VB 20%	2.93 ± 0.08^ab^	0.27 ± 0.03^bc^	0.54 ± 0.03^b^	0.40 ± 0.05^b^
QB 10%	1.98 ± 0.04^d^	0.27 ± 0.04^bc^	0.49 ± 0.02^c^	0.48 ± 0.02^a^
QB 20%	3.06 ± 0.08^a^	0.23 ± 0.05^c^	0.62 ± 0.03^a^	0.47 ± 0.03^a^
PB 10%	2.17 ± 0.06^c^	0.35 ± 0.07^ab^	0.51 ± 0.02^bc^	0.48 ± 0.03^a^
PB 20%	2.84 ± 0.14^b^	0.39 ± 0.05^a^	0.56 ± 0.02^ab^	0.40 ± 0.06^b^

Leg.: WF-wheat flour; VF-*Verde* fava bean flour; QF-*Quelcao* fava bean flour; PF-*Peruanita* fava bean flour; CB-control bread (100% wheat flour); VB-bread with partial replacement (10 or 20%) of wheat flour with *Verde* fava bean flour; QB-bread with partial replacement of wheat flour with *Quelcao* fava bean flour; PB-bread with partial replacement of wheat flour with *Peruanita* fava bean flour. GAE-gallic acid equivalents; CE-catechin equivalents. Results are expressed as mean ± standard deviation on a fresh weight basis. Different letters within the same column, either among flours or among breads, indicate statistically significant differences according to analysis of variance (ANOVA) (*p* ≤ 0.05), followed by Tukey’s test (*p* ≤ 0.05)

The ABTS•+ assay values for Peruvian faba bean flours were higher than those reported for faba beans by Mitic et al. [12] cultivated in Serbia and by Ceramella et al. [13] grown in Southern Italy, but similar to those reported by Salvador-Reyes et al. [2] for Peruvian faba beans. In the DPPH assay, *Quelcao* and *Peruanita* faba bean flours exhibited an antioxidant potential approximately four times higher than that of wheat flour, and also higher than those reported by Mitic et al. [12], but comparable to the values reported by Valente et al. [14] for faba beans cultivated in Europe.

The total phenolic content in Peruvian faba bean flours was significantly higher than in wheat flour, with the *Peruanita* cultivar presenting the highest content (1.61 ± 0.08 mg GAE/g), approximately five times greater than that of wheat flour. However, these values were lower than those reported in other studies for faba bean seeds from different regions extracted with methanol [12, 14, 15], suggesting that such variations may be attributed to differences in extraction methods, cultivation conditions, and varieties.

The flavonoid content in Peruvian faba bean flours was approximately five times higher than in wheat flour, although the observed values were lower than those reported in some studies [16, 17]. Nevertheless, they were higher than those found by Boudjou et al. [18] and Zhu et al. [19]. These findings highlight the potential of faba bean flours as valuable sources of antioxidants and bioactive compounds, suggesting that their incorporation into food formulations may enhance the nutritional quality and oxidative stability of products.

The differences observed in antioxidant potential, total phenolic content, and total flavonoid content among the studied Peruvian fava bean cultivars may be attributed to cultivar-specific characteristics, including genetic background, seed size, pigmentation, and matrix composition [2, 10, 12, 14]. Seed pigmentation, in particular, has been associated with a higher accumulation of phenolic compounds, especially flavonoids and tannins, which contribute to antioxidant activity [14–16, 18]. In addition, variations in seed coat thickness and cellular structure may influence phenolic localization and extractability [12, 18]. Similar genotype-dependent variability in phenolic composition and antioxidant behavior has been reported for fava beans and other legumes, highlighting the importance of cultivar-level investigations when assessing the functional properties of legume-derived ingredients [12, 15, 17].

### Antioxidant Potential, Total Phenolic Content (TPC), and Total Flavonoid Content (TFC) in Breads Made with Raw Peruvian Faba Bean Flours from Different Cultivars

Partial substitution of wheat flour with faba bean flour from the *Verde*, *Quelcao*, and *Peruanita* cultivars resulted in breads with higher antioxidant potential compared to the control bread, as measured by the ABTS•+ assay (Table 1). In the DPPH assay, only breads containing 10 and 20% *Peruanita* faba bean flour showed values significantly higher than the control. Breads containing 20% faba bean flour from the *Verde* and *Quelcao* cultivars exhibited the highest antioxidant potential in the ABTS•+ assay, with VB 20% and PB 20% breads showing a significantly higher potential than the control bread (1.25 ± 0.10 µmol ET/g). In the DPPH assay, breads with 10 and 20% *Peruanita* faba bean flour demonstrated the highest antioxidant potential (0.39 ± 0.05 µmol ET/g), exceeding that of the other breads, but lower than formulations in which 20% of wheat flour was completely replaced with a mixture of pea flour and green bean flour or mesquite flour and green bean flour [20].

The analysis of total phenolics showed that all breads with partial substitution exhibited higher values than the control. Breads QB20% and PB20% displayed the highest total phenolic contents (0.62 ± 0.03 and 0.56 ± 0.02 mg GAE/g, respectively), followed by VB20% and PB10% (0.54 ± 0.03 and 0.51 ± 0.02 mg GAE/g). These values were lower than those reported for bread formulations with partial substitution of whole wheat flour by mixtures of different legumes [20] and for baked crackers with 40% wheat flour replaced by pulse flours [21]. However, in general, the total phenolic content was higher than that found in breads with wheat flour partially replaced by lupin (*Lupinus albus*) flour at 10%, similar to those with 20% substitution, and lower than formulations with 30% substitution [22].

Regarding flavonoid content, breads QB10%, QB20%, and PB10% showed significantly higher values than the control, with the lowest content observed in VB10% (0.37 ± 0.03 mg CE/g). The highest total flavonoid contents were found in QB10%, QB20%, and PB10% (0.48 ± 0.02, 0.47 ± 0.03 and 0.47 ± 0.03 mg CE/g, respectively). These values exceeded those reported in breads with wheat flour replaced by lupin (*Lupinus albus*) flour at levels ranging from 10 to 30% [22].

The addition of Peruvian bean flours to bread formulations significantly enhances antioxidant potential as well as total phenolic and flavonoid contents, which may confer health benefits by protecting against oxidative damage and reducing the risk of diseases associated with oxidative stress [14, 23].

### Effect of *In Vitro* Digestion on the Antioxidant Potential and Bioaccessibility of Total Phenolic Content (TPC) in Raw Flours of Peruvian Beans from Different Cultivars

The antioxidant potential of Peruvian fava bean flours from different cultivars (*Verde*, *Quelcao*, and *Peruanita*) increased after digestion compared to wheat flour, as measured by the ABTS•+ and DPPH radical scavenging assays. At the end of digestion, no significant differences in antioxidant potential were observed among the fava bean cultivars using the ABTS•+ method. Pellegrini et al. [24] reported a significant increase in the antioxidant potential of chia seeds following digestion, suggesting that the release of bioactive compounds and their chemical transformations may contribute to this phenomenon. Using the DPPH method, *Peruanita* fava bean flour exhibited the highest antioxidant potential (18.43 ± 2.39 µmol TE/g), followed by the *Verde* and *Quelcao* cultivars (13.52 ± 1.53 µmol TE/g and 14.77 ± 2.97 µmol TE/g, respectively) (Table 2). Li et al. [25] reported similar increases in antioxidant activity after digestion, highlighting that intestinal digestion can enhance antioxidant capacity due to the release of bioactive phenolic compounds. Furthermore, protein digestion may generate bioactive peptides with antioxidant potential [23].

**Table 2:** Bioaccessibility of total phenolic content (TPC) and the effect of simulated gastrointestinal digestion on the antioxidant potential of *Peruvian fava* bean flours from different cultivars

Sample	Antioxidant potential (µmol TE/g)		TPC (mg EAG/g)
	ABTS	DPPH	
WF	1.33 ± 0.09^c^	0.22 ± 0.03^d^	0.30 ± 0.02^c^
D-WF	2.87 ± 0.09^c^	2.49 ± 0.45^c^	2.67 ± 0.38^b^
D/UD	2.16	11.32	8.90
VF	23.78 ± 0.99^b^	0.55 ± 0.04^c^	1.40 ± 0.09^b^
D-VF	56.78 ± 7.85^a^	13.52 ± 1.53^b^	4.60 ± 0.95^a^
D/UD	2.39	24.58	3.26
QF	24.05 ± 0.73^b^	0.77 ± 0.54^b^	1.50 ± 0.09^b^
D-QF	55.73 ± 10.02^a^	14.77 ± 2.97^b^	4.36 ± 0.84^a^
D/UD	2.32	19.18	2.91
PF	26.13 ± 0.93^a^	0.90 ± 0.10^a^	1.61 ± 0.08^a^
D-PF	56.17 ± 5.33^a^	18.43 ± 2.39^a^	4.70 ± 0.89^a^
D/UD	2.15	20.48	2.92

CB-control bread; VF-*Verde* fava bean flour; QF-*Quelcao* fava bean flour; PF-*Peruanita* fava bean flour; D-simulated gastrointestinal digestion; UD-undigested; GAE-gallic acid equivalent; CE-catechin equivalent. Results are expressed as mean ± standard deviation on a fresh weight basis. Different letters within the same column indicate statistically significant differences according to Analysis of Variance (ANOVA, *p* ≤ 0.05), followed by Tukey’s *post hoc* test (*p* ≤ 0.05)

The greater increase in antioxidant potential observed with the DPPH method, compared to ABTS•+, may be attributed to the higher sensitivity of DPPH to hydrophobic antioxidants released from the food matrix during digestion. Perez-Perez et al. [26] suggested that hydrolysis of phenolic compounds during digestion may enhance their bioaccessibility and antioxidant capacity, facilitating intestinal absorption. Fava bean flours also exhibited higher total phenolic content after digestion, consistent with observations in other legumes [25, 27].

Gastric and intestinal digestion appear to increase both the abundance and release of different forms of polyphenols due to pH changes and enzymatic activity, thereby reducing interactions between phenolic compounds and carbohydrates. These findings indicate that digestion can improve the availability of antioxidants in fava bean flours, potentially offering health benefits by mitigating oxidative stress [25].

### Bioaccessibility of Total Phenolic Content (TPC) and the Effect of In Vitro Digestion on the Antioxidant Potential of Breads

The antioxidant potential of the breads increased after in vitro digestion, as measured by the ABTS•+ and DPPH assays. However, breads with a 20% substitution of wheat flour with faba bean flour (*Verde*, *Quelcao*, and *Peruanita*) did not show a significant increase in the ABTS•+ assay, whereas all breads exhibited higher values in the DPPH assay after digestion. In the ABTS•+ assay, the control breads and those containing 10% faba bean flour (VB10%, QB10%) did not differ significantly from each other and showed lower values than breads containing 20% faba bean flour (QB20%, PB10%, PB20%) (Table 3). These results are consistent with those reported by Szawara-Nowak et al. [28], who observed an increase in the antioxidant activity of breads after digestion, with breads enriched with buckwheat flour exhibiting higher ABTS radical-scavenging capacity.

**Table 3:** Bioaccessibility of total phenolics and the effect of simulated gastrointestinal digestion on the antioxidant potential of breads produced with Peruvian fava bean flours from different cultivars

Sample	Antioxidant potential (µmol TE/g)		TPC (mg EAG/g)
	ABTS	DPPH	
CB	1.25 ± 0.10^e^	0.21 ± 0.04^c^	0.35 ± 0.07^d^
D-CB	1.79 ± 0.34^e^	1.44 ± 0.62^f^	2.34 ± 0.16^ab^
D/UD	1.4	6.86	6.68
VB 10%	1.87 ± 0.12^dD^	0.20 ± 0.10^cH^	0.49 ± 0.04^cD^
D-VB 10%	2.08 ± 0.25^eCD^	1.61 ± 0.16^eE^	1.86 ± 0.32^cABC^
D/UD	1.11	8.05	3.79
VB 20%	2.93 ± 0.08^abAB^	0.27 ± 0.03^bcFGH^	0.54 ± 0.03^bD^
D-VB 20%	2.73 ± 0.33^deCD^	2.24 ± 0.06^cC^	2.17 ± 0.39^abcABC^
D/UD	0.93	8.30	4.02
QB 10%	1.98 ± 0.04^dCD^	0.27 ± 0.04^bcFGH^	0.49 ± 0.02^cD^
D-QB 10%	2.04 ± 0.31^deCD^	1.88 ± 0.05^dD^	1.89 ± 0.27^bcBC^
D/UD	1.03	6.96	3.86
QB 20%	3.06 ± 0.08^aA^	0.23 ± 0.04^cGH^	0.62 ± 0.03^aD^
D-QB 20%	2.56 ± 0.13^bcB^	2.60 ± 0.16^bB^	2.54 ± 0.46^aA^
D/UD	0.84	11.30	4.10
PB 10%	2.17 ± 0.06^cC^	0.35 ± 0.06^aFG^	0.51 ± 0.02^bcD^
D-PB 10%	2.23 ± 0.12^cdC^	2.12 ± 0.04^cC^	2.34 ± 0.41^abABC^
D/UD	1.03	6.06	4.59
PB 20%	2.84 ± 0.14^bAB^	0.39 ± 0.05^aF^	0.56 ± 0.02^abD^
D-PB 20%	2.24 ± 0.10^cdC^	2.96 ± 0.05^aA^	2.37 ± 0.55^abAB^
D/UD	0.79	7.59	4.23

CB-control bread (100% wheat flour); VB-bread with partial substitution (10 or 20%) of wheat flour with *Verde* fava bean flour; QB-bread with partial substitution of wheat flour with *Quelcao* fava bean flour; PB-bread with partial substitution of wheat flour with *Peruanita* fava bean flour; D-simulated gastrointestinal digestion; UD-undigested; GAE-gallic acid equivalent; CE-catechin equivalent. Results are expressed as mean ± standard deviation on a fresh weight basis. Different lowercase letters (including all formulations) and uppercase letters (excluding the control bread–CB) within the same column indicate statistically significant differences according to analysis of variance (ANOVA) at *p* ≤ 0.05, followed by Tukey’s *post hoc* test at *p* ≤ 0.05

In the DPPH assay, all breads exhibited a significant increase in antioxidant potential post-digestion, with PB20% bread showing the highest value (2.96 ± 0.05 µmol ET/g) and VB10% bread the lowest (1.61 ± 0.16 µmol ET/g). Chait et al. [29] similarly reported a significant increase in the antioxidant activity of carob following *in vitro* digestion, consistent with the observations of the present study.

Total phenolic contents increased after digestion in all breads, with no significant differences between the control breads and those containing VB20%, QB20%, PB10%, and PB20%. However, breads VB10% and QB10% exhibited lower values. Lafarga et al. [27] reported significant increases in total phenolics after* in vitro* digestion of breads enriched with broccoli and buckwheat flours, respectively.

*In vitro* digestion induces the release of phenolic compounds, possibly due to changes in pH and enzymatic activity. Swieca et al. [30] highlighted that wheat flour contains a high content of bound phenolics, which are released during digestion. The bioaccessibility and bioavailability of TPC can vary depending on digestive conditions, such as pH and enzymatic activity [31]. Baublis et al. [32] observed that digestion can release bound phenolic acids, thereby increasing the antioxidant capacity of food products. Furthermore, digestion can generate bioactive peptides with potential antioxidant properties [27]. The increase in total phenolics may result from the release of conjugated compounds during digestion, where chemical structure and food matrix interactions influence bioaccessibility [33].

Although cereal–legume composite flours have been extensively investigated [4, 5, 20–22, 28] the present study contributes new evidence by focusing on underutilized Peruvian fava bean cultivars and by integrating baking and *in vitro* gastrointestinal digestion to assess antioxidant potential and phenolic bioaccessibility. Rather than providing compound-level identification, this work adopts a functional screening approach to evaluate digestion-induced changes in antioxidant activity in both flours and breads. The consistent increase in antioxidant potential and phenolic bioaccessibility after digestion highlights the relevance of food matrix interactions and digestive processes in modulating the functional properties of composite bakery products. The absence of phenolic profiling represents a limitation; however, it also defines a clear direction for future studies aimed at elucidating structure–function relationships using chromatographic techniques.

## Conclusion

This study provides functional evidence supporting the use of underutilized Peruvian fava bean cultivars in bread formulations based on refined wheat flour. Although cereal–legume compositing is a well-established strategy, the results demonstrate that partial substitution of refined wheat flour with raw Peruvian fava bean flours enhance antioxidant potential and phenolic bioaccessibility after *in vitro* gastrointestinal digestion. These findings highlight the relevance of considering digestive processes when assessing the functional quality of composite foods. While the present study was not designed to elucidate compound-specific or enzymatic mechanisms, it offers meaningful insights into the functional performance of standard bread formulations enriched with Andean legumes. Future studies comparing refined and whole wheat formulations, as well as incorporating detailed phenolic profiling and mechanistic approaches, are warranted to further elucidate the interactions between cereal–legume matrices and their implications for functional and nutritional quality.

## Supplementary Information

Below is the link to the electronic supplementary material.

Supplementary Material 1

## Data Availability

Data will be made available on request.
