# Supplementary material for: Functional Potential of Peruvian Fava Bean Flours in Bread: Antioxidant Activity and Phenolic Bioaccessibility
Source: Plant Foods Hum Nutr. 2026 Mar 21;81(2):37. doi: 10.1007/s11130-026-01487-z (PMC13005775; doi:10.1007/s11130-026-01487-z)
Supplement: Supplementary file 1 — Supplementary Material 1 [file 11130_2026_1487_MOESM1_ESM.docx]

**Supplementary Material**

2.1 Sample preparation

Flours from three cultivars of Peruvian fava beans—*Verde*, *Quelcao*, and *Peruanita*—were used (Figure 1). The seed lots correspond to the same materials previously characterized in our earlier study [1]. The seeds were imported from Peru as dry grains (registration numbers: E0000113N GACSMR, E0000213N GACSMR, and E0000313N GACSMR for *Verde*, *Quelcao*, and *Peruanita*, respectively), and the flours were produced at the Department of Food Science and Nutrition, University of Campinas (Unicamp), following the protocol described below:


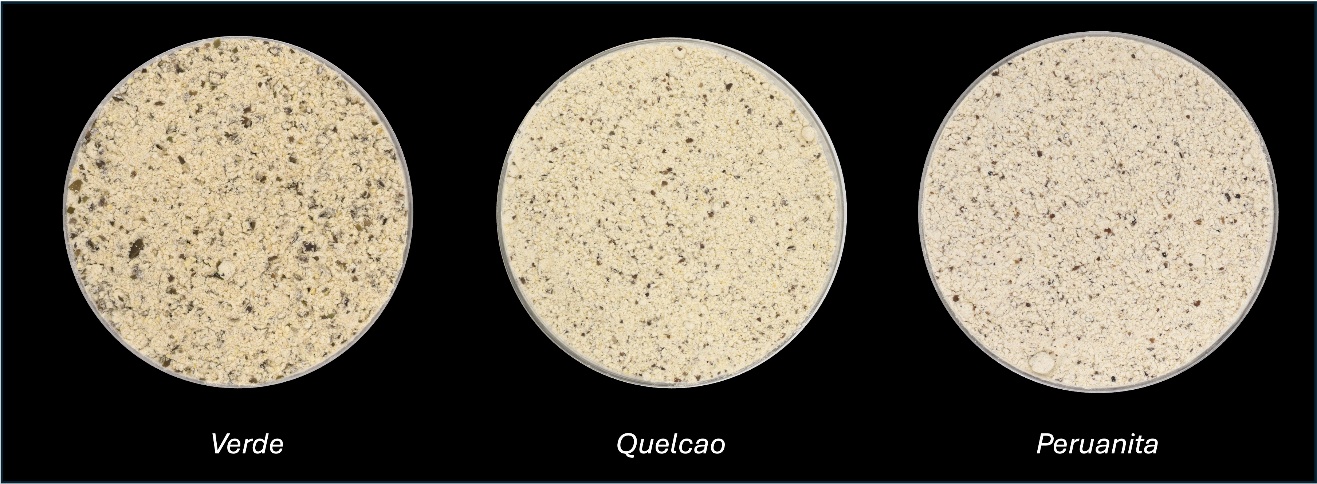


Figure 1 - Flours from three cultivars of Peruvian fava beans, *Verde*, *Quelcao*, and *Peruanita*.

The beans were soaked in distilled water (1:10, w/v) for 24 h at room temperature. For flour production, the soaked beans were dried in a forced-air oven at 50 °C for 24 h until reaching 10% moisture content and subsequently ground in a hammer mill.

The flours were packaged separately by cultivar type and sent to the Food Technology Laboratory of the Federal University of Alfenas (UNIFAL-MG), where they were stored at –20 °C until use for extract preparation and bread production.

Six different loaf-type bread formulations were developed using raw fava bean flours (*Verde*, *Quelcao*, and *Peruanita*) as partial substitutes (10% or 20%) for wheat flour (3 cultivars × 2 substitution ratios = 6 bread formulations) (Table 1).

Table 1. Formulations of mixed flours from peruvian fava beans and wheat

| Formulation | Wheat flour (%) | Peruvian fava bean flour (%) | | |
| --- | --- | --- | --- | --- |
|  |  | *Verde* | *Quelcao* | *Perunaita* |
| Control (CB) | 100 | - | - | - |
| *Verde* 10% (VB10%) | 90 | 10 | - | - |
| *Verde* 20% (VB20%) | 80 | 20 | - | - |
| *Quelcao* 10% (QB10%) | 90 | - | 10 | - |
| *Quelcao* 20% (QB20%) | 80 | - | 20 | - |
| *Peruanita* 10% (PB10%) | 90 | - | - | 10 |
| *Peruanita* 20% (PB20%) | 80 | - | - | 20 |

Preliminary trials were conducted to define appropriate substitution levels of wheat flour with Peruvian fava bean flours. Substitution levels above 20% negatively affected dough development and bread volume. Therefore, substitution levels of 10% and 20% (w/w) were selected as a balance between functional enhancement and technological feasibility, in agreement with literature reports indicating that partial replacement of wheat flour with legume flours, including fava bean, typically ranges from 5% to 20% without compromising product quality [2, 3]. All formulations were compared with a control prepared exclusively with 100% refined white wheat flour (Figure 2).


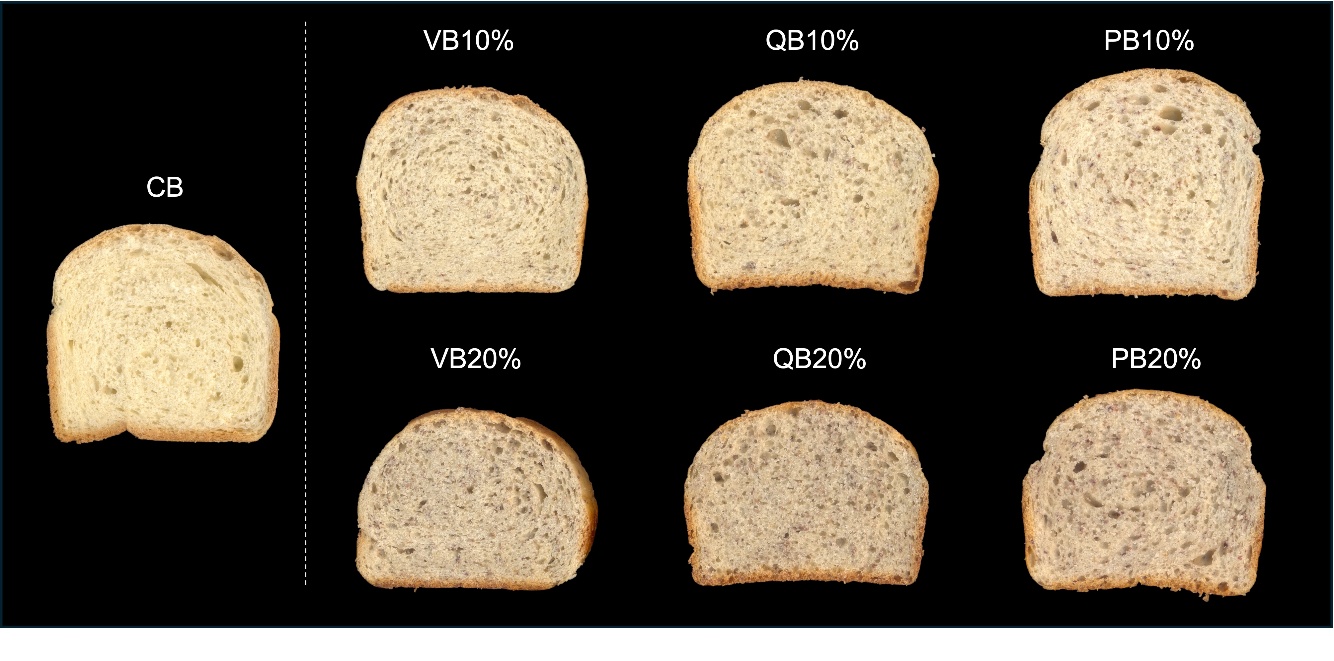


Figure 2 - Bread formulations with partial substitution (10% and 20%) of wheat flour with Peruvian fava bean flours (*Verde*, *Quelcao*, and *Peruanita*) compared with the control bread (100% wheat flour).

The proportions of the remaining ingredients in all formulations were as follows: 67.6% refined white wheat flour (control bread) or a mixture containing 10% or 20% fava bean flour blended with white wheat flour; 11.2% sugar, 11.2% vegetable fat, 6.7% powdered milk, 2.2% instant yeast, and 1.1% salt. Except for the fava bean flours, all other ingredients were purchased from local retailers in Alfenas, Minas Gerais, Brazil.

Bread loaves were produced and baked in triplicate for each formulation using a Cadence-Finezza bread-making machine (model PAD 502) at the Food Technology Laboratory of the Federal University of Alfenas (UNIFAL-MG), following the basic 500 g cycle with a light crust setting. After baking, the loaves were cooled on a wire rack at room temperature for 1 h 30 min. Following cooling, the breads were photographed to capture top and side views, sliced in half, packaged, and the halves were frozen. Each sample was labeled and stored under freezing conditions until extract preparation.

2.2 Extract preparation

For extraction, the breads were finely grated and stored in a freezer until use. Two different extracts were obtained for each sample using ethanol (99.9%) and water (4:1 v/v) as solvents, following the procedure described by Verardo et al. [4] with modifications. Approximately 0.5 g of grated bread was weighed in triplicate into 15 mL Falcon tubes. To each tube, 5 mL of solvent solution was added, and the mixture was homogenized for 60 s using a benchtop vortex mixer (Global Trade Technology XH-DU, Jaboticabal, São Paulo, Brazil). The mixture was then placed in an ultrasonic bath at 40 kHz and 25 °C for 30 min, followed by an additional 30 s of vortex homogenization. Subsequently, the mixture was centrifuged (Eppendorf SE, Centrifuge 5810 R, Barkhausenweg, Hamburg, Germany) at 4000 × g for 10 min at 4 °C, and the supernatant was collected. These steps were repeated twice more, each time adding 5 mL of solvent. Finally, the supernatants were combined, and the total volume was recorded. The filtered extracts were then subjected to antioxidant potential analysis using the ABTS•⁺ and DPPH assays, as well as total phenolic and flavonoid contents determination by spectrophotometry.

2.3 Determination of antioxidant potential (ABTS and DPPH method), total phenolic content (TPC) and total flavonoid content (TFC)

The antioxidant potential was determined using the spectrophotometric method based on the discoloration of the ABTS•⁺ cationic radical (2,2′-azinobis(3-ethylbenzothiazoline-6-sulfonic acid)), resulting from its neutralization by antioxidant compounds present in the samples [5, 6]. The ABTS•⁺ solution was prepared by mixing a 7 mM aqueous ABTS•⁺ solution with a 2.45 mM potassium persulfate solution, followed by incubation in the dark at room temperature for 12 h. The resulting solution was then diluted until reaching an absorbance of 0.70 ± 0.05, measured using a BelPhotonics Ultraviolet/Visible-M51 spectrophotometer (Monza, Milan, Italy) set at a wavelength of 734 nm.

The color difference between the reading of the fully colored solution, considered as 100% ABTS•⁺ free radicals (750 μL of ABTS•⁺ + 250 μL of 70% v/v ethanol solution), and the reading of the ABTS•⁺ radical solution containing the sample extract (750 μL of ABTS•⁺ + 250 μL of appropriately diluted sample) was measured after incubation in the dark for 15 min. The mixture was analyzed using a spectrophotometer at 734 nm. The results were compared with an analytical calibration curve constructed with seven points corresponding to different concentrations (1–17 nmol/mL, R^2^ = 0.992) of a Trolox (6-hydroxy-2,5,7,8-tetramethylchroman-2-carboxylic acid) standard solution. Two blank solutions were prepared by replacing the sample aliquot with water while maintaining the ABTS•⁺ solution. Absorbance values obtained from each sample were corrected by subtracting the corresponding blank solution readings, which exhibited the highest absorbance values. Results were expressed as micromoles of Trolox equivalent per gram (µmol TE/g).

The DPPH (2,2-diphenyl-1-picrylhydrazyl) radical-scavenging assay was conducted according to the method described by Salvador-Reyes et al. [1]. Aliquots of suitably dilute samples (100 µl) were added to a test tube along with 650 µl of a stock solution of 2,2-diphenyl-1-picrylhydrazyl (DPPH; 0.1 mM) prepared in methanol. The samples were incubated in a dark environment for 30 min, and the absorbance was measured at 517 nm at 25 °C, using the same extractor solution as a negative control. The standard curves were prepared with different concentrations of Trolox (1.0–14.7 nmol/ml, R^2^ = 0.981).

The total phenolic content of the samples was determined according to the method described by Salvador-Reyes et al. [1], based on Folin and Denis [7]. An aliquot of 160 µL of the properly diluted sample, obtained from the extract, was added to a test tube containing 80 µL of the Folin–Ciocalteu reagent and manually agitated for 10 s. Subsequently, 640 µL of Na₂CO₃ solution (7.5% w/v) was added, and the mixture was incubated in the dark at 25 °C for 30 min. Absorbance was measured at 756 nm using a spectrophotometer. Quantification was performed using the linear regression equation obtained from a standard curve constructed with seven points, corresponding to different concentrations of gallic acid standard (1–10 µg/mL, R^2^ = 0.0989). Results were expressed as milligrams of gallic acid equivalents per gram of sample (mg GAE/g).

The total flavonoid content was determined according to the methodology previously described by Christ and Müller [8], with modifications [1]. An aliquot of 625 µL of the properly diluted sample was added to a test tube. Subsequently, 37.5 µL of sodium nitrite (NaNO₂) solution (5% w/v) was added, and the mixture was manually agitated for approximately 10 s and incubated for 5 min at 25 °C. After incubation, 37.5 µL of aluminum chloride (AlCl₃) solution (10% w/v) was added. The mixture was manually agitated for 10 s and incubated again for 5 min at 25 °C. Then, 250 µL of sodium hydroxide (NaOH) solution (1 M) and 350 µL of distilled water were added, and the tubes were manually agitated and incubated under the same conditions as previously described. Absorbance was measured using a spectrophotometer at a wavelength of 510 nm. A 10-point calibration curve with catechin standard (4.5-46 µg/mL, R^2^ = 0.995) was constructed and used for quantification, and the results were expressed as milligrams of catechin equivalents per gram of sample (mg CE/g).

2.4 Bioaccessibility of total phenolic and flavonoid contents (TPC and TFC) and the effect of in vitro digestion on antioxidant potential

Gastrointestinal digestion was performed according to the INFOGEST protocol [9, 10] with minor modifications. The procedure simulated the entire gastrointestinal digestion process: oral, gastric, and intestinal phases.

In the oral phase, 5 g of previously grated fava bean bread sample was mixed in a 50 mL Falcon tube with 4 mL of simulated salivary fluid [KCl, KH₂PO₄, NaHCO₃, MgCl₂(H₂O)₆, (NH₄)₂CO₃, HCl], 25 µL of 0.3 M CaCl₂(H₂O)₂, and 975 µL of salivary amylase solution to achieve a final enzymatic activity of 75 U/mL, considering a final sample and simulated fluid volume of 10 mL. The ratio of bread sample to simulated salivary fluid was 1:1 (w/w). The mixture was incubated at 37 °C under agitation (50 rpm) for 2 min.

During the gastric phase, the entire oral-phase solution was adjusted to pH 3.0 using 300 μL of 1 M HCl and mixed with 8.5 mL of simulated gastric fluid [KCl, KH₂PO₄, NaHCO₃, NaCl, MgCl₂(H₂O)₆, (NH₄)₂CO₃, HCl], 5 µL of 0.3 M CaCl₂(H₂O)₂, and 1,195 µL of pepsin to reach a final enzymatic activity of 2,000 U/mL in a total volume of 20 mL. The mixture was incubated at 37 °C under agitation (50 rpm) for 2 h.

In the intestinal phase, the entire gastric digestion solution was adjusted to pH 7.0 using 450 μL of 1 M NaOH and combined with 11.51 mL of simulated intestinal fluid [KCl, KH₂PO₄, NaHCO₃, NaCl, MgCl₂(H₂O)₆, (NH₄)₂CO₃, HCl], 40 µL of 0.3 M CaCl₂(H₂O)₂, 3 mL of bile salt solution, and 5 mL of pancreatin solution to achieve final bile concentration and trypsin activity in pancreatin of 10 mM and 100 U/mL, respectively, in a total volume of 40 mL. The mixture was incubated at 37 °C under agitation (50 rpm) for 2 h. Enzymatic activity was stopped at the end of the *in vitro* digestion by immersing the test tube in an ice bath.

The final mixture was centrifuged at 7,000 × g for 10 min at 4 °C (Eppendorf SE centrifuge, Model 5810 R, Hamburg, Germany). The supernatant was then collected, filtered, and stored in Eppendorf tubes at −80 °C until antioxidant potential, total phenolic content, and total flavonoid content analyses. The digestion protocol was carried out in triplicate. Control treatments were performed without the addition of the respective enzymes but including bile salts, all simulated fluids, and maintaining identical pH, temperature, and agitation conditions.

2.8 Statistical analysis of data

All determinations of antioxidant potential, total phenolic contents, and total flavonoid contents were performed in triplicate. Results are expressed as mean ± standard deviation on a fresh weight (*fw*). To assess the existence of significant differences among the stages of *in vitro* digestion, data were subjected to Analysis of Variance (ANOVA), followed by Tukey’s test (p < 0.05) to identify which groups differed from each other, using the statistical software Minitab® version 16.2.3.

**References**

1. Salvador-Reyes R., Furlan LC, Martínez-Villaluenga C, Dala-Paula B M, Nabeshima EH, Pinto CC, Souza SMde, Pallone JAL, & Clerici MTPS (2024) Peruvian fava beans for health and food innovation: physicochemical, morphological, nutritional, and techno-functional characterization. Food Res. Int. 192, 114814. <https://doi.org/10.1016/j.foodres.2024.114814>

2. Levy BR (2014) La biblia del pan (1ª ed.). Barcelona, Espanha: Ediciones Paidós.

3. Udomkun P, Masso C, Swennen R, Romuli S, Innawong B, Fotso Kuate A, Akin-Idowu PE, Alakonya A & Vanlauwe B (2022) Comparative study of physicochemical, nutritional, phytochemical, and sensory properties of bread with plantain and soy flours partly replacing wheat flour. Food Science & Nutrition 10: 3085–3097. <https://doi.org/10.1002/fsn3.2907>

4. Verardo V, Glicerina V, Cocci E, Frenich AG, Romani S, & Caboni MF (2018) Determination of free and bound phenolic compounds and their antioxidant activity in buckwheat bread loaf, crust, and crumb*.* LWT - Food Sci. Technol. 87, 217-224. <https://doi.org/10.1016/j.lwt.2017.08.063>.

5. Re R, Pellegrini N, Proteggente A, Pannala A, Yang M, & Rice-Evans C (1999) Antioxidant activity applying an improved ABTS radical cation decolorization assay. *Free Radic. Biol. Med.* 26(9–10), 1231–1237. <https://doi.org/10.1016/S0891-5849(98)00315-3>

6. Brand-Williams W, Cuvelier ME, & Berset C (1995) Use of free-radical method to evaluate antioxidant activity. *LWT - Food Sci. Technol.* 28, 25–30. <https://doi.org/10.1016/S0023-6438(95)80008-5>

7. Folin O, & Denis W (1912) On phosphotungstic-phosphomolybdic compounds as color reagents. *JBC* 12(2), 239–243. <https://doi.org/10.1016/S0021-9258(18)88697-5>

8. Christ B, & Müller KH (1960) Zur serienm¨aßigen Bestimmung des Gehaltes an Flavonol-Derivaten in Drogen. *Archiv der Pharmazie* 293(12), 1033–1042. <https://doi.org/10.1002/ardp.19602931202>

9. Brodkorb A, Egger L, Alminger M, Alvito P, … & Recio I (2019) INFOGEST static *in vitro* simulation of gastrointestinal food digestion. Nature Protocols 14(4), 991-1014. <https://doi.org/10.1038/s41596-018-0119-1>.

10. Minekus M, Alminger M, Alvito P, Ballance S, Bohn T, Bourlieu C, ... & Brodkorb A (2014) A standardised static *in vitro* digestion method suitable for food—An international consensus. Food Funct. 5, 1113-1124. [https://doi.org/10.1039/C3FO60702J](https://doi.org/10.1039/C3FO60702J). Acesso em: 22 maio 2023.
